# Differential responses to high- and low-dose ultraviolet-B stress in tobacco Bright Yellow-2 cells

**DOI:** 10.3389/fpls.2015.00254

**Published:** 2015-04-21

**Authors:** Shinya Takahashi, Kei H. Kojo, Natsumaro Kutsuna, Masaki Endo, Seiichi Toki, Hiroko Isoda, Seiichiro Hasezawa

**Affiliations:** ^1^Department of Integrated Biosciences, Graduated School of Frontier Sciences, The University of TokyoKashiwa, Japan; ^2^Alliance for Research on North Africa, University of TsukubaTsukuba, Japan; ^3^Ph. D. Program in Life Science Innovation, University of TsukubaTsukuba, Japan; ^4^LPixel Inc.Bunkyo-ku, Japan; ^5^Plant Genome Engineering Research Unit, Agrogenomics Research Center, National Institute of Agrobiological SciencesTsukuba, Japan

**Keywords:** BY-2, cell cycle, cell death, checkpoint, DNA damage, ultraviolet-B

## Abstract

Ultraviolet (UV)-B irradiation leads to DNA damage, cell cycle arrest, growth inhibition, and cell death. To evaluate the UV-B stress–induced changes in plant cells, we developed a model system based on tobacco Bright Yellow-2 (BY-2) cells. Both low-dose UV-B (low UV-B: 740 J m^−2^) and high-dose UV-B (high UV-B: 2960 J m^−2^) inhibited cell proliferation and induced cell death; these effects were more pronounced at high UV-B. Flow cytometry showed cell cycle arrest within 1 day after UV-B irradiation; neither low- nor high-UV-B–irradiated cells entered mitosis within 12 h. Cell cycle progression was gradually restored in low-UV-B–irradiated cells but not in high-UV-B–irradiated cells. UV-A irradiation, which activates cyclobutane pyrimidine dimer (CPD) photolyase, reduced inhibition of cell proliferation by low but not high UV-B and suppressed high-UV-B–induced cell death. UV-B induced CPD formation in a dose-dependent manner. The amounts of CPDs decreased gradually within 3 days in low-UV-B–irradiated cells, but remained elevated after 3 days in high-UV-B–irradiated cells. Low UV-B slightly increased the number of DNA single-strand breaks detected by the comet assay at 1 day after irradiation, and then decreased at 2 and 3 days after irradiation. High UV-B increased DNA fragmentation detected by the terminal deoxynucleotidyl transferase dUTP nick end labeling assay 1 and 3 days after irradiation. Caffeine, an inhibitor of ataxia telangiectasia mutated (ATM) and ataxia telangiectasia and Rad3-related (ATR) checkpoint kinases, reduced the rate of cell death in high-UV-B–irradiated cells. Our data suggest that low-UV-B–induced CPDs and/or DNA strand-breaks inhibit DNA replication and proliferation of BY-2 cells, whereas larger contents of high-UV-B–induced CPDs and/or DNA strand-breaks lead to cell death.

## Introduction

Ultraviolet (UV)-B radiation (280–320 nm), a component of sunlight, is unavoidable for plants because of their sessile life. This radiation may lead to growth inhibition or even cell death. UV-B induces formation of pyrimidine photodimers, such as cyclobutane pyrimidine dimers (CPDs) and pyrimidine (6-4) pyrimidone photoproducts, and thus inhibits DNA replication and transcription, increases the number of mutations, and induces cell cycle arrest and cell death (Lo et al., 2005; de Lima-Bessa et al., 2008). Higher plants have multiple DNA repair mechanisms (Mannuss et al., 2012). For example, under light, the UV-B-induced photodimers are repaired by photolyases specific to each photodimer; mammals have no homologous genes (Ahmad et al., 1997; Nakajima et al., 1998; Hidema et al., 2000; Takeuchi et al., 2007). Photolyases are activated by UV-A/blue light (Hada et al., 2000; Teranishi et al., 2008). The dark repair involves nucleotide excision repair and base excision repair mechanisms (Mannuss et al., 2012). Severe damage by ionizing radiation and UV-B radiation may also result in generation of DNA strand breaks, which are repaired by the homologous recombination, non-homologous end joining, and microhomology-mediated end joining systems (Ries et al., 2000; Amiard et al., 2013). Endoreduplication in response to UV-B irradiation has been reported; whether it may play a protective role in UV-B tolerance is unknown (Radziejwoski et al., 2011).

In mammals, UV-B radiation interferes with cell cycle progression (Garinis et al., 2005; Ortolan and Menck, 2013). In Arabidopsis, photodimer formation causes cell cycle arrest and inhibits hypocotyl elongation (Biever et al., 2014). Arabidopsis shares several DNA damage checkpoint mechanisms with mammals and yeast (Yoshiyama et al., 2013b). The phosphatidyl-3-kinase family members, ataxia telangiectasia mutated (ATM) and ataxia telangiectasia and Rad3-related (ATR) kinases, are required for initiation of DNA damage responses (DDRs) (Roy, 2014). ATM mainly responds to DNA double-strand break (DSB) induced by ionizing radiation and chemical mutagens (Garcia et al., 2003). ATR mainly responds to single-stranded DNA (ssDNA) and replication stressors, such as hydroxyurea, aphidicolin, and UV stress (Culligan et al., 2004). In Arabidopsis, ATR, and its partner protein ATRIP are involved in tolerance to UV-B stress (Culligan et al., 2004; Sakamoto et al., 2009). Mammalian ATR and ATM phosphorylate checkpoint kinases 1 and 2 (CHK1 and CHK2) and activate the p53 transcription factor (Cimprich and Cortez, 2008; Shiloh and Ziv, 2013). However, plants have no homologs of p53, CHK1, or CHK2. In Arabidopsis, suppressor of gamma 1 (SOG1) functions in genotoxic stress–induced cell death and checkpoint mechanisms (Yoshiyama et al., 2009, 2013a; Adachi et al., 2011), and in UV-B–induced programmed cell death and growth retardation (Furukawa et al., 2010; Biever et al., 2014).

Although inhibition of cell cycle progression by UV-B in Arabidopsis root cells synchronized with hydroxyurea has been reported (Jiang et al., 2011), the details of UV-B-induced production of photodimers and their role in growth inhibition, cell cycle arrest, and cell death in higher plants remain unclear, in part because of the lack of appropriate experimental models.

Tobacco bright yellow-2 (BY-2) cells are non-green because they lack developed chloroplasts. They are larger than Arabidopsis cells, have high proliferation rates (80–100-fold per week) and are easy to synchronize for cell cycle progression studies (Nagata et al., 1992; Kumagai-Sano et al., 2006). BY-2 cells have also been used to study responses to pathogens, oxidative stress, and genotoxic stress, including UV stress (Perennes et al., 1999; Kadota et al., 2005; Sano et al., 2006; Lytvyn et al., 2010; Smetana et al., 2012). They are highly suitable for observations of cell death and organelle alterations in response to stresses (Higaki et al., 2007).

In this study, to investigate cellular responses to UV-B irradiation, we developed a model using BY-2 cells. Low-dose UV-B irradiation inhibited cell proliferation and induced cell death with low frequency, whereas high doses induced cell death. This difference may have been caused by differences in the amounts of UV-B–induced photodimers, DNA strand breaks, or both. We also found that ATM, ATR, or both kinases mediated UV-B stress–induced cell death.

## Materials and methods

### Plant material and culture conditions

Tobacco BY-2 (*Nicotiana tabacum* L. cv. Bright Yellow 2) suspension-cultured cells were maintained by weekly dilution (1:95) with modified Linsmaier and Skoog (LS) medium as described by Kumagai-Sano et al. (2006). Cell suspensions were agitated on a rotary shaker at 130 rpm at 27°C in the dark.

### UV treatments

A UV-B fluorescent lamp (FL20SE; Kyokko Denki, Japan, Supplemental Figure 1) was used. Seven day-old BY-2 cells were diluted (1:40) with LS medium (Perennes et al., 1999) and incubated as above for 1 h; 10 mL of cell suspension was transferred into a plastic Petri dish, covered with a UV29 quartz glass filter (cut-off of <290 nm; Hoya Glass, Japan) (Ioki et al., 2008), and exposed to 1.6 W m^−2^ of UV-B for up to 31 min. In some experiments, immediately after UV-B irradiation, UV-A (18.3 W m^−2^) was supplied by a UV-A fluorescent lamp (FL20S-BL; Toshiba, Japan, Supplemental Figure 1) through the UV29 quartz glass filter for 30 min. After irradiation, BY-2 cells were transferred to a flask and cultured with agitation under standard conditions. The intensities of UV-B and UV-A irradiation were measured by a MS-211-I UV photometer with a sensor specific to the UV-B and UV-A lamp spectrum (EKO Instruments, Japan).

### Fresh weight determination

A 1-mL aliquot of cell suspension were transferred to microtubes and centrifuged for 30 s at 5000 rpm. Supernatants were removed by aspiration and pellets were weighed in at least three independent experiments.

### Dead cell counting

Dead cells were detected by the Evans blue method as described by Ohno et al. (2011). In brief, cells from a 1-mL aliquot of suspension were collected by centrifugation, incubated with 0.05% Evans blue (Wako, Japan) for 10 min and then washed with water. Dead cells (stained blue) were counted under a microscope (BX51; Olympus, Japan). At least 500 cells were counted in each experiment.

### Flow cytometry

Flow cytometry was performed as described by Ohno et al. (2011). Frozen BY-2 cell pellets were chopped in extraction buffer with a sharp razor blade to extract the nuclei, filtered through 30-μm filters; isolated nuclei were stained with a CyStain UV Precise P kit (Partec, Germany). DNA content was determined with a Ploidy Analyzer (Partec).

### Synchronization of BY-2 cells and determination of mitotic index

BY-2 cells were synchronized as described by Kumagai-Sano et al. (2006). Mitotic index was determined by counting 4′, 6-Diamidino-2-phenylindole, dihydrochloride (DAPI) stained nuclei using a fluorescence microscope (BX51). At least 300 cells were counted in each experiment.

### DNA extraction and detection of UV-induced CPD formation by ELISA

Total genomic DNA was extracted from frozen BY-2 cell pellets using DNeasy Plant Mini Kit (QIAGEN, CA) and samples were diluted to 0.5 μg mL^−1^ with phosphate buffered saline (PBS) buffer. CPD formation was measured by enzyme-linked immuno-sorbent assay (ELISA) as previously described (Takeuchi et al., 1996; Takahashi et al., 2002) with slight modifications. Commercial monoclonal antibody BM12 (1:5000; Kyowa Medex Co., Japan) and ECL Anti-mouse IgG, Horseradish Peroxidase-linked Whole antibody (from sheep) (GE Healthcare, UK) were used and absorbance was measured at 492 nm by using a microplate reader (Viento nano; DS Pharma Biomedical, Japan).

### Detection of DNA strand breaks by comet assay

Comet assay was performed as described by Menke et al. (2001) with modifications. In brief, frozen BY-2 cell pellets were chopped in PBS buffer with a razor blade to release the nuclei. The nuclei were filtered through the 30-μm filters, mixed with Comet LM agarose, applied to CometSlide (CometAssay kit; Trevigen Inc., Germany) on a heating block at 42°C; slides were incubated for 15 min at 4°C in the dark. The number of single-strand breaks was measured according to the alkaline/neutral (A/N) protocol (Menke et al., 2001). Slides were incubated in 0.3 M NaOH, 5 mM EDTA (pH 13.5) for 10 min at room temperature, equilibrated with 1× TBE buffer (3 × 5 min) at 4°C in the dark. Electrophoresis (25 V, 6 mA) was performed at room temperature in 1× TBE for 20 min. Then the slides were soaked in 1% Triton X-100 for 10 min, 70% EtOH (2 × 5 min), 99.5% EtOH (2 × 5 min), dried at 37°C for 30 min and stained with SYBR Green Gold Nucleic Acid cell stain (Invitrogen-Life Technologies, CA). Images were captured using a fluorescence microscope (BX51). Comet tails were quantified using the KBI plug-in “Cometassay” for ImageJ software (http://hasezawa.ib.k.u-tokyo.ac.jp/zp/Kbi/ImageJKbiPlugins).

### Detection of DNA fragmentation by TUNEL assay

Terminal deoxynucleotidyl transferase dUTP nick-end labeling (TUNEL) assay was performed according to the manufacturer's protocol (*In Situ* Cell Death Detection Kit, Fluorescein; Roche Diagnostics GmbH, Germany) with modifications. Samples were fixed in 4% paraformaldehyde buffered with PBS for 20 min at 4°C, and labeled with fluorescein. Fluorescein signals and DAPI-stained cells were detected by using a fluorescence microscope.

### Caffeine treatment

Cells were treated with caffeine as described by Smetana et al. (2012). In brief, BY-2 cells were pretreated with 5 mM caffeine for 1 h, irradiated with high UV-B, cultured for 24 h, and the dead cells were counted as described above.

### Statistical analysis

Student's *t*-test in Microsoft Excel 2007 software was used for statistical analysis.

## Results

### Effect of UV-B irradiation on BY-2 cell proliferation and death

**Figure 1:**
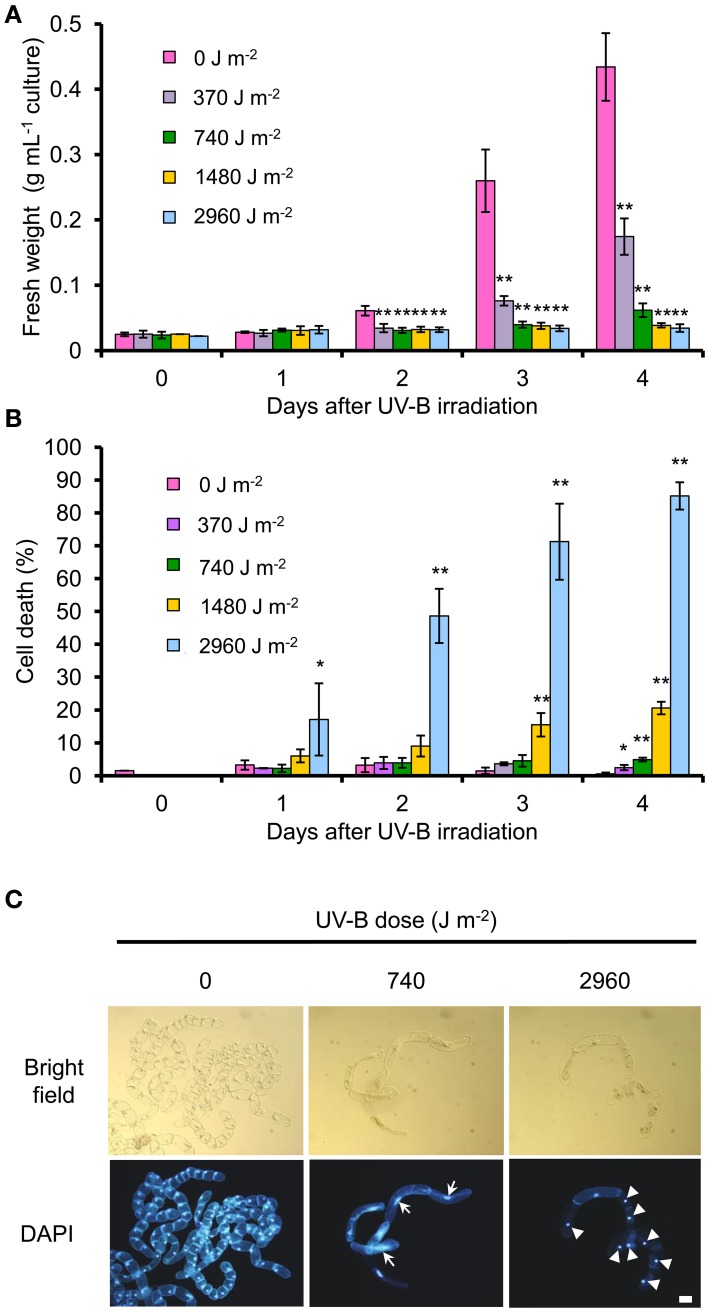
**Effect of UV-B radiation on cell proliferation and cell death**. BY-2 cells were irradiated with UV-B (0–2960 J m^−2^) and cultured for 4 days. The cells (1 mL) were centrifuged and the supernatant discarded. **(A)** Fresh weight of the collected cells was measured. **(B)** Evans blue staining was used to quantify dead cells. Results from at least two independent experiments are shown. Error bars show SD. Asterisks indicate significant differences from control (no UV-B) (*P < 0.05, **P < 0.01). **(C)** Bright-field images and DAPI staining of control cells (no UV-B), or cells irradiated with low UV-B (740 J m^−2^) or high UV-B (2960 J m^−2^). Arrows indicate enlarged cells with elongated nuclei. Arrowheads indicate putative condensed nuclei. Bar = 50 μm.

We determined how four different UV-B doses affect BY-2 cell proliferation and cell death. As an indicator of cell proliferation, we used fresh weight of centrifuged cell pellets. Cell proliferation was inhibited at 2 days after irradiation at all UV-B doses tested (Figure 1A). At 4 days after irradiation, the fresh weight of cells irradiated at 2960, 1480, and 740 J m^−2^ was <15% of that of non-irradiated cells, whereas the fresh weight of cells irradiated at 370 J m^−2^ was 41% of that of the control cells (Figure 1A). The number of dead cells reached 17% after UV-B irradiation at 2960 J m^−2^ at 1 day. At 4 days, it was 85% at 2960 J m^−2^, 20.6% at 1480 J m^−2^, and <10% at 740 J m^−2^ and 370 J m^−2^ (Figure 1B).

Thus, we set two UV-B conditions, low UV-B (740 J m^−2^) to inhibit cell proliferation, and high UV-B (2960 J m^−2^) to inhibit cell proliferation and induce cell death. Low UV-B resulted in only a small number of dead cells but led to the enlargement of the cells and nuclei (Figure 1C). Cell enlargement and nuclear elongation may be caused by increasing the nuclear DNA content above 4C (Yasuhara and Kitamoto, 2014). Low UV-B–irradiated cells did not reach the nuclear DNA content of 8C (data not shown). High UV-B irradiation led to cytoplasm shrinkage and nuclear condensation in dead cells (Figure 1C).

### UV-B irradiation induces cell cycle arrest in BY-2 cells

**Figure 2:**
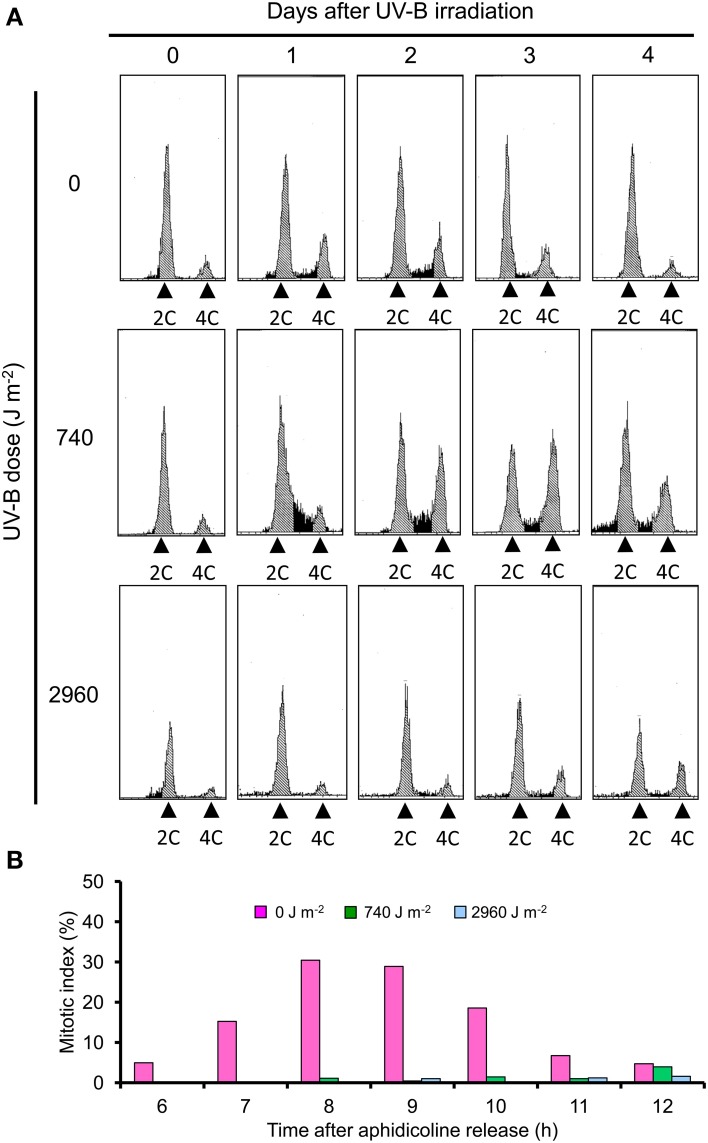
**Analysis of cell cycle progression in UV-B–irradiated BY-2 cells. (A)** Cells irradiated with UV-B (0, 740, or 2960 J m^−2^) were analyzed by flow cytometry. DNA ploidy distribution was measured with a ploidy analyzer (Partec, Germany). Typical data from at least two independent experiments are shown. **(B)** BY-2 cells were synchronized at S phase by aphidicolin and irradiated with UV-B (0, 740, or 2960 J m^−2^) after release. Mitotic index was determined at indicated time points. Typical data from at least two independent experiments are shown.

The DNA content of most BY-2 cells in the stationary phase was 2C, indicating that most cells were at G1 phase before UV-B irradiation. At 1 day after low UV-B irradiation, the cell cycle was arrested at the 2C–4C transition (G1/S transition). At 3–4 days after UV-B irradiation, the number of cells at 4C (G2/M phase) increased but transition from S to G2/M phase and from G2/M to G1 was slower than that of non-irradiated cells (Figure 2A). After high UV-B irradiation, almost all cells were arrested at 2C at 1 or 2 days, and only a small number of cells reached 4C (Figure 2A).

Next, we synchronized the cells at the S phase by adding aphidicolin. In control cells, the mitotic index peaked at 7–9 h after aphidicolin release (Figure 2B). In low and high UV-B–irradiated cells, no clear peak of mitotic index was detected within 12 h after aphidicolin release (Figure 2B). Thus, we concluded that low and high UV-B irradiation induced cell cycle arrest at G1 or S phase; cell cycle arrest at G2/M may be also induced at 3–4 days after low UV-B irradiation.

### UV-A irradiation suppresses changes induced by low UV-B and cell death induced by high UV-B irradiation

**Figure 3:**
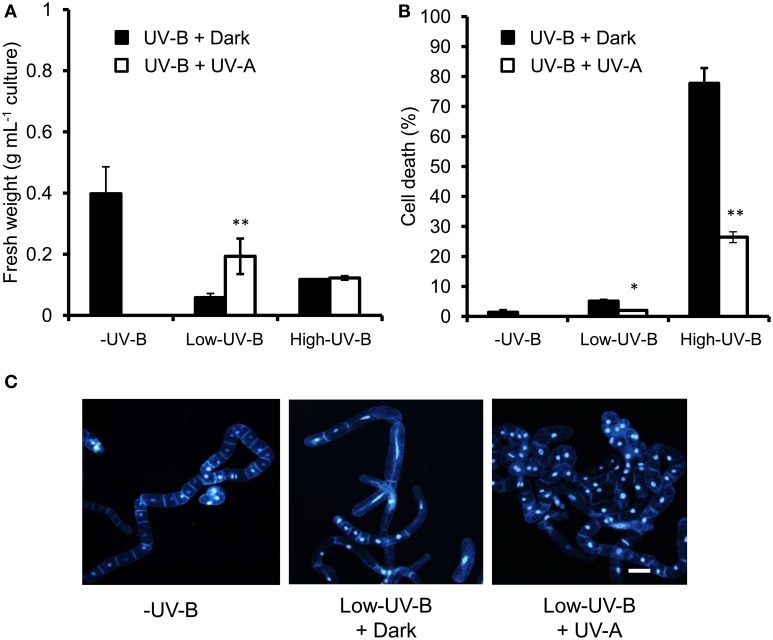
**Effects of UV-A radiation on UV-B-induced changes in cell proliferation and cell death**. BY-2 cells were irradiated with UV-B (0, 740, or 2960 J m^−2^), followed by 30-min incubation in the dark (UV-B + Dark) or 30 min UV-A irradiation (UVB + UVA). **(A)** Fresh weight; **(B)** the percentage of dead cells. Error bars in **(A,B)** show SD (*n* = 3). Asterisks indicate significant differences from the UV-B + Dark treatment (*P < 0.05, **P < 0.01). **(C)** DAPI-stained cells at 4 d after irradiation. Bar = 50 μm.

To examine the possibility of repair of UV-B-induced damage by activating a photolyase, we checked the effects of UV-A irradiation. A 30-min exposure to UV-A radiation immediately after UV-B irradiation partially prevented inhibition of cell proliferation induced by low UV-B (Figure 3A) and cell death induced by low and high UV-B (Figure 3B). The number of low UV-B–irradiated cells with enlarged nuclei and increased cell volume decreased at 4 days after UV-A irradiation (Figure 3C).

### UV-B irradiation induces CPD formation, which may be reduced by UV-A irradiation

**Figure 4:**
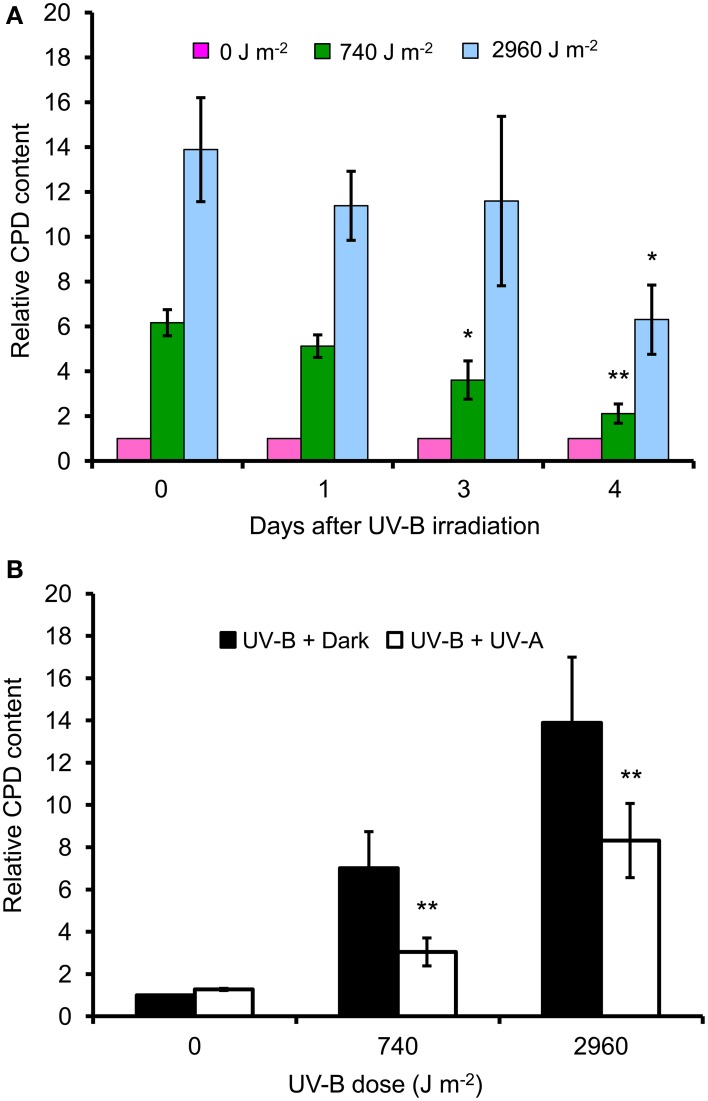
**Accumulation of cyclobutane pyrimidine dimers in UV-B–irradiated cells and reduction by UV-A radiation**. Contents of cyclobutane pyrimidine dimers (CPDs) were measured by ELISA with CPD-specific antibody (BM12; Kyowa Medex Co., Japan). **(A)** The contents of CPD at 0, 1, 3, and 4 days after UV-B irradiation relative to that in non-irradiated cells. Error bars show SD (*n* = 3). Asterisks indicate significant differences from the non-irradiated control (*P < 0.05, **P < 0.01). **(B)** Amounts of CPDs in cells irradiated with UV-A immediately after UV-B exposure at indicated doses (0, 740, or 2960 J m^−2^) relative to those in cells irradiated only with UV-B at 0 days after irradiation. Error bars show SD (*n* = 3). Asterisks indicate significant differences from the UV-B + Dark treatment (*P < 0.05, **P < 0.01).

Immediately after irradiation, the CPD amounts in high UV-B–irradiated cells were approximately twice those in low UV-B–irradiated cells. In low UV-B–irradiated cells, the CPD content decreased by approximately 66% at 4 days after irradiation. In high UV-B–irradiated cells, the CPD content decreased by 52% at 4 days after irradiation (Figure 4A, Supplemental Figure 2).

The CPD content in cells exposed to UV-A following low UV-B irradiation was decreased by half in comparison with control cells. In high UV-B–irradiated cells, the CPD content was also significantly reduced by UV-A irradiation and reached the level observed in low UV-B–irradiated cells without UV-A irradiation (Figure 4B).

### UV-B irradiation induces DNA strand breaks in BY-2 cells

**Figure 5:**
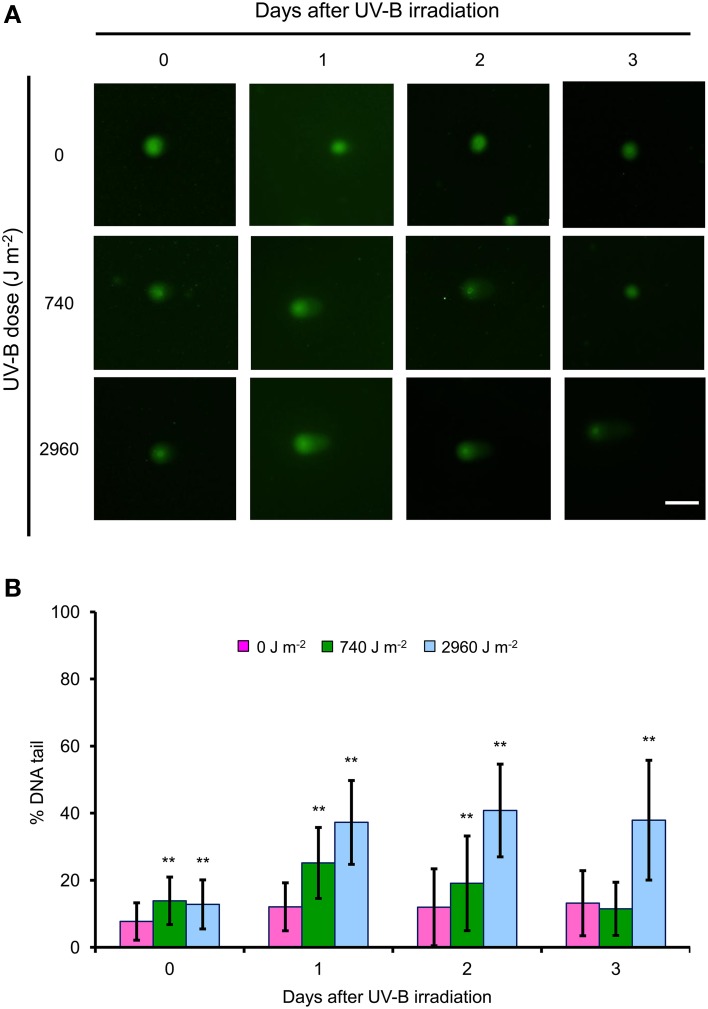
**Single-strand breaks induced by low- and high-dose UV-B irradiation**. Frequencies of DNA strand breaks were measured by the comet assay (Menke et al., 2001). BY-2 cells were irradiated with UV-B (0, 740, or 2960 J m^−2^) and cultured for 3 days. Nuclei were assayed by using the alkaline/neutral protocol, which detects mainly single-strand breaks (Menke et al., 2001). **(A)** SYBR Green–stained nuclei at 1, 2, and 3 days after 0, 740, and 2960 J m^−2^ of UV-B irradiation. Bar = 100 μm. **(B)** DNA strand breaks were quantified by using the KBI plug-in “Cometassay” for ImageJ software and expressed as percentage of DNA in the tail. Error bars show SD. Asterisks indicate significant differences from control (no UV-B) (*P* < 0.01). Typical results from two independent experiments are shown.

**Figure 6:**
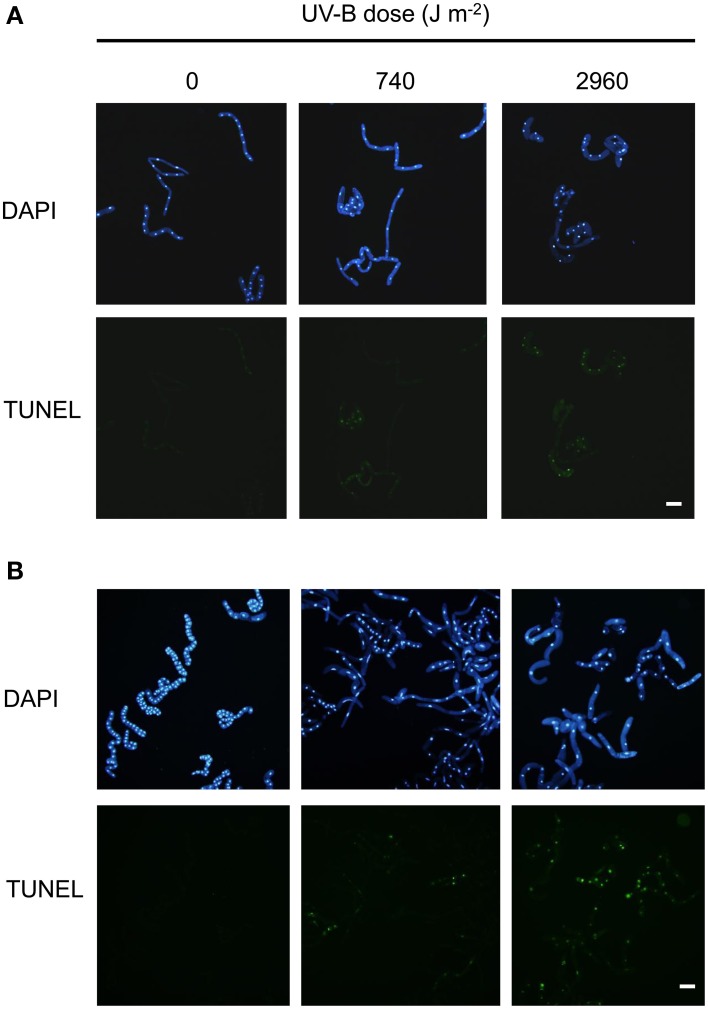
**Fragmented DNA in cells irradiated with low- and high-dose UV-B**. BY-2 cells were irradiated with UV-B (0, 740, or 2960 J m^−2^) and cultured for **(A)** 1 day or **(B)** 3 days. DNA fragmentation was analyzed by TUNEL assay. Fluorescein signals (TUNEL) and DAPI staining are shown. Bars = 100 μm.

Immediately after irradiation with low or high UV-B, the number of single-strand breaks increased significantly. Single-strand breaks transiently increased at 1 day after low UV-B irradiation, and then gradually decreased at 2–3 days, whereas they continued to increase over 3 days after high UV-B irradiation (Figure 5). TUNEL assay detects fluorescent labeling of DNA strand breaks by using terminal deoxynucleotidyl transferase (TdT), which catalyzes the polymerization of labeled nucleotides to free 3′-OH DNA ends in a template-independent manner (Gavrieli et al., 1992). The TUNEL assay is often used to detect DNA fragmentation, including single-strand breaks and DSBs (Kwon et al., 2013). At 1 and 3 days after irradiation, TUNEL signals were detected in both low and high UV-B irradiated cells; the signals were observed more frequently in high UV-B–irradiated cells than in low UV-B–irradiated cells (Figure 6). These results suggest that low and high UV-B irradiation induces the single-strand breaks, and may cause DSB formation.

### High UV-B irradiation induces ATM and ATR kinase–mediated cell cycle arrest and/or cell death in BY-2 cells

**Figure 7:**
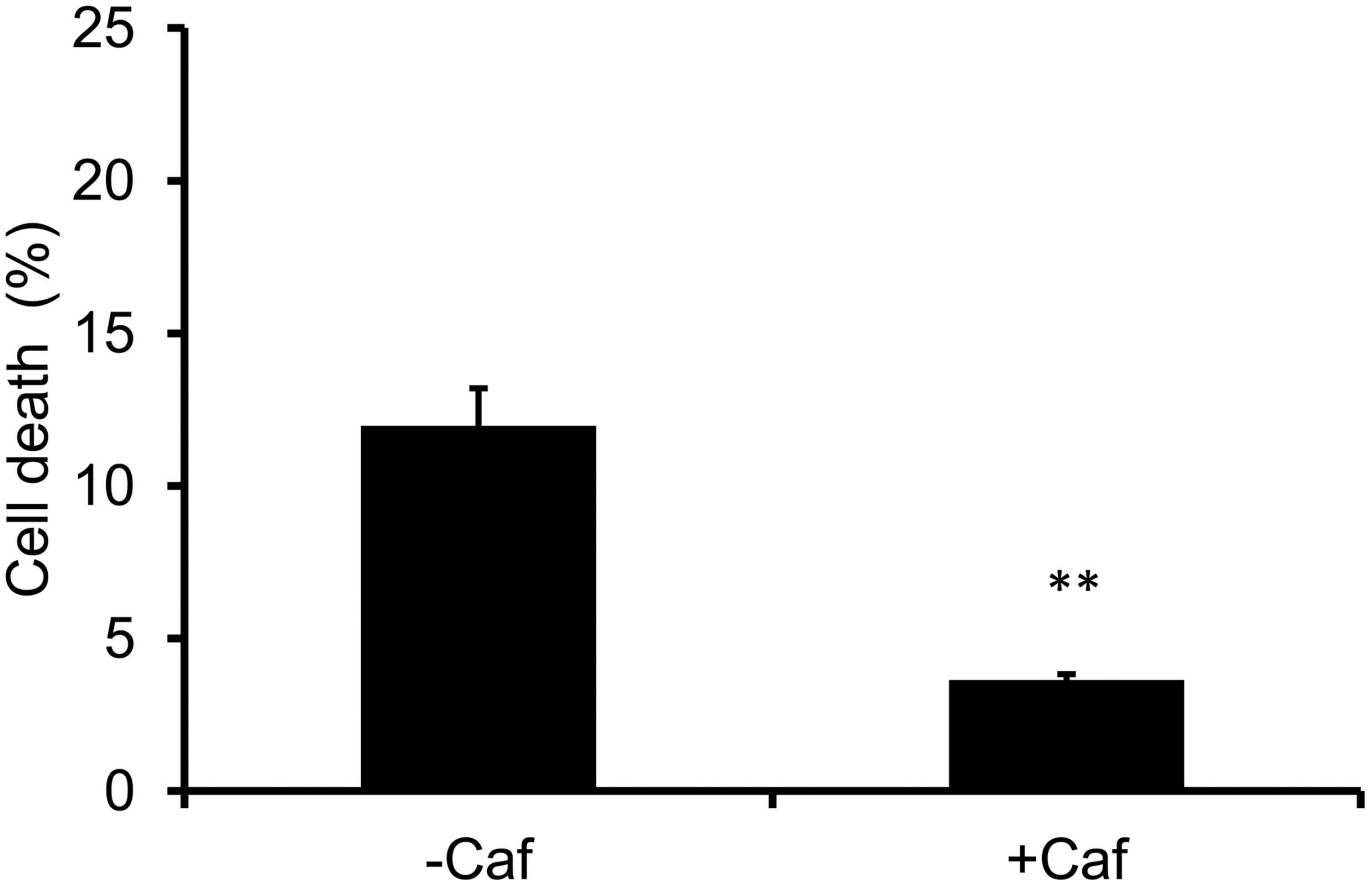
**Caffeine reduces cell death induced by high-dose UV-B**. BY-2 cells pretreated with 5 mM caffeine (+Caf) or without caffeine (–Caf) for 1 h were exposed to UV-B (2960 J m^−2^) and cultured for 24 h. Dead cells stained with Evans blue were counted. Error bars show SD (*n* = 3). Asterisks indicate a significant difference from –Caf (*P* < 0.01).

To confirm the roles of ATM and ATR kinases in the UV-B–induced cell death, we used their inhibitor, caffeine. Pretreatment with caffeine for 1 h followed by high UV-B irradiation reduced cell death at 24 h (Figure 7). This result indicates that checkpoint signaling mediated by ATM, ATR, or both kinases is involved in high UV-B–induced cell death.

## Discussion

UV-B radiation induces cell cycle arrest and cell death, yet how DNA damage triggers cell cycle arrest and cell death in plant cells remains unclear. In this study, we investigated the dose-dependence of inhibition of cell proliferation and induction of cell death by UV-B in BY-2 cells.

### BY-2 cells irradiated with low and high UV-B show different damage responses

Low UV-B inhibited cell proliferation and induced cell death with low frequency. The low UV-B–induced CPDs were formed but declined within several days. The UV-A exposure immediately after low UV-B irradiation prevented inhibition of cell proliferation and reduced the CPD content. High UV-B induced inhibition of cell proliferation and cell death. High UV-B induced larger amounts of CPDs than low UV-B, and they declined although somewhat slower. The UV-A exposure immediately after high UV-B irradiation prevented cell death but did not prevent inhibition of cell proliferation. These results suggest a strong relationship between CPD formation and dose-dependent inhibition of cell proliferation and induction of cell death by UV-B.

In low UV-B–irradiated cells, CPDs were detected until 3–4 days, but cell proliferation and cell cycle progression were observed. In plants, translesion DNA synthesis is involved in UV-B tolerance (Sakamoto et al., 2003; Takahashi et al., 2005; Anderson et al., 2008). Translesion DNA synthesis–type DNA polymerases bypass DNA lesions and allow DNA synthesis to progress with DNA lesions. This mechanism might be functional in BY-2 cells.

### Low UV-B–induced CPDs may generate transient single-strand breaks and induce cell cycle arrest

Low UV-B irradiation led to transient single-strand breaks and cell cycle arrest during the G1 and S phases at 1–2 days. The amounts of CPDs and single-strand breaks were reduced and cell cycle progression restarted at 3–4 days after UV-B irradiation. In Arabidopsis, the ATR-mediated pathway provides tolerance to UV-B stress (Culligan et al., 2004; Sakamoto et al., 2009). DNA integrity may be restored by ATR recruitment and DNA lesion bypass activity (Furukawa et al., 2010; Curtis and Hays, 2011). The ATR-mediated pathway might be activated by the presence of single-strand breaks and trigger cell cycle arrest in our system.

In addition to cell cycle arrest during the G1 and S phases at 1–2 days after UV-B irradiation, many cells were arrested at the G2/M checkpoint at 2–3 days after UV-B irradiation. In Arabidopsis roots, UV-B downregulates the expression of *Histone H4* and *E2Fa* (cell cycle progression markers), delays *CYCD3;1* (a positive factor in G1-to-S transition) expression, and upregulates the expression of *KRP2* (a negative regulator of the G1-to-S transition), suggesting that UV-B irradiation induces the G1-to-S arrest (Jiang et al., 2011). Molecular mechanisms of UV-B–induced cell cycle arrest are still mostly unknown. It is interesting to analyze the temporal expression of G1/S or G2/M-specific marker genes after UV-B irradiation.

### High UV-B induces large CPD amounts, persistent single-strand breaks, permanent cell cycle arrest, and high rate of cell death

High UV-B irradiation induced CPDs (twice as many as low UV-B did), single-strand breaks, and permanent cell cycle arrest at the G1/S transition. CPD accumulation may induce single-strand breaks (and eventually DSBs), and prolonged presence of CPDs or single-strand breaks may accelerate cell death (Garinis et al., 2005; Lopes et al., 2006). In Arabidopsis root tip stem-cell niches, programmed cell death induced by roughly 30,000 unrepaired photoadducts generated by UV-B irradiation was similar to that caused by 24 DSBs generated by gamma radiation (Furukawa et al., 2010). In Arabidopsis, the entry into S phase in the presence of ssDNA may generate DSBs due to collapse of DNA replication forks (Curtis and Hays, 2011). Thus, high UV-B may induce not only CPDs and single-strand breaks but DSB formation, and trigger cell death.

Among high UV-B–irradiated cells, TUNEL-positive cells were observed from the next day after irradiation; fewer TUNEL-positive cells were observed upon low UV-B irradiation. Bleomycin-treated BY-2 cells show paraptosis-type cell death without DNA fragmentation (Smetana et al., 2012), whereas BY-2 cells exposed to very high UV-B (283 kJ m^−2^) show apoptosis-type programmed cell death that involves DNA fragmentation (Lytvyn et al., 2010). High UV-B–induced cell death observed in this study is likely apoptosis-like programmed cell death.

### Checkpoint kinases, ATM and ATR, may regulate UV-B-induced cell death

In our study, caffeine treatment reduced high UV-B–induced cell death. UV-B may activate both ATR-mediated and ATM-mediated pathways (Furukawa et al., 2010). Because caffeine inhibits both ATR and ATM pathways, their relative contributions are unclear. In mammals, caffeine inhibits UV-B mediated ATR/Chk1 pathways and decreases the levels and phosphorylation of Cyclin B1, which mediates entry to mitosis (Conney et al., 2013). Whether this also takes place in plants is unclear. In the future, we will need to investigate which pathway contributes most to UV-B–induced cell death in BY-2 cells.

ATM and ATR kinase–mediated DDRs regulate programmed cell death (Fulcher and Sablowski, 2009). In Arabidopsis DDR mutants, UV-B–induced DNA strand breaks trigger ATM/ATR/SOG1–mediated cell death (Furukawa et al., 2010; Curtis and Hays, 2011). In this study, we directly observed the formation of UV-B–induced CPDs and transient formation of single-strand breaks. Their content may affect the survival vs. cell death choice in UV-B–irradiated BY-2 cells; at least, our data show that the ATM/ATR pathways mediate high UV-B–induced cell death and thus provide evidence for DDR-mediated UV-B–induced cell death in BY-2 cells.

### Advantages and disadvantages of the BY-2 cell model for studying UV-B responses

In green tissues, it may often be difficult to interpret the effects of UV-B on growth retardation because UV-B also affects photosynthesis (Allen et al., 1997; Takeuchi et al., 2002). Thus, the potential effects of UV-B on the cell cycle or signal transduction are difficult to distinguish from its direct effects on photosynthesis. As BY-2 cells are non-green, they do not pose these problems in studies of UV-B responses.

Non-green tissues, such as those of etiolated seedlings, might offer an advantage for investigations of photoregulation of light-responsive genes (Gardner et al., 2009). UV-B activates the phenylpropanoid pathway, which includes phenylalanine ammonia-lyase and chalcone synthase (CHS), and it serves to synthesize UV-B–absorbing compounds such as flavonoids (Kusano et al., 2011). This mechanism is mediated by the UV-B–specific receptor UV-resistance 8 (UVR8) (Brown and Jenkins, 2008; Biever et al., 2014). It would be interesting to investigate the physiological roles of the UV-B–inducible flavonoid biosynthesis pathway in regulation of the cell cycle by using the BY-2 model system if the *CHS* and *UVR8* genes are expressed in these cells.

Few studies of UV-B effects on plant cellular components are available (Lytvyn et al., 2010). In mammalian cells, UV-B induces apoptosis by a mechanism that involves caspase-8 activation and mitochondrial dysfunction (Takasawa et al., 2005). BY-2 cells are also good experimental tools for studying organelles, including mitochondria (Arimura et al., 2004; Sano et al., 2005; Higaki et al., 2007); it would be interesting to investigate UV-B–induced changes in subcellular compartments to clarify the mechanisms leading to cell death in these cells.

Although an expressed sequence tag clone library of BY-2 cells has been established (Matsuoka et al., 2004; Gális et al., 2006), the complete tobacco genome sequence (unlike the Arabidopsis genome sequence) has not been available until recently, and this has limited molecular analysis in BY-2 cells. In 2014, the draft genome sequence of *Nicotiana tabacum*, which is the source of the BY-2 line, was published (Sierro et al., 2014). In future, this information, coupled with use of the BY-2 cell model, may help to clarify in detail how UV-B affects plant cells, from the initial signaling events to cellular responses to UV-B irradiation.

In this study, we used an experimental model to investigate temporal changes in UV-B–induced damage responses in BY-2 cells. Our results suggest that DNA damage mediates UV-B dose–dependent responses that affect cell cycle regulation and cell death.

### Conflict of interest statement

The authors declare that the research was conducted in the absence of any commercial or financial relationships that could be construed as a potential conflict of interest.
